# Partial loss of VE-cadherin improves long-term outcome and cerebral blood flow after transient brain ischemia in mice

**DOI:** 10.1186/s12883-016-0670-8

**Published:** 2016-08-18

**Authors:** Karen Gertz, Golo Kronenberg, Ria Uhlemann, Vincent Prinz, Ruben Marquina, Monica Corada, Elisabetta Dejana, Matthias Endres

**Affiliations:** 1 Klinik und Hochschulambulanz für Neurologie, Charité Universitätsmedizin Berlin, Charitéplatz 1, 10117 Berlin, Germany; 2 Center for Stroke Research Berlin (CSB), Charité Universitätsmedizin Berlin, Charitéplatz 1, 10117 Berlin, Germany; 3 Klinik und Hochschulambulanz für Psychiatrie und Psychotherapie, Charité Universitätsmedizin Berlin, Charité Campus Mitte, Charitéplatz 1, 10117 Berlin, Germany; 4 Klinik für Neurochirurgie, Charité Universitätsmedizin Berlin, Campus Virchow Klinikum, Augustenburger Platz 1, 13353 Berlin, Germany; 5 IFOM, FIRC institute of Molecular Oncology, Via Adamello 16, 20139 Milan, Italy; 6 Department of Biosciences, University of Milan, Via Celoria 26, 20133 Milan, Italy; 7 Department of Immunology, Genetics and Pathology, Rudbeck Laboratory, Uppsala University, 75185 Uppsala, Sweden; 8 Excellence Cluster NeuroCure, Charitéplatz 1, 10117 Berlin, Germany; 9 German Center for Neurodegenerative Disease (DZNE), Charitéplatz 1, 10117 Berlin, Germany; 10 German Centre for Cardiovascular Research (DZHK), Charitéplatz 1, 10117 Berlin, Germany

**Keywords:** Cerebral ischemia, Stroke, Endothelium, Pericyte, Adhesion molecule, Angiogenesis

## Abstract

**Background:**

VE-cadherin is the chief constituent of endothelial adherens junctions. However, the role of VE-cadherin in the pathogenesis of cerebrovascular diseases including brain ischemia has not yet been investigated.

**Methods:**

VE-cadherin heterozygous (VEC^+/-^) mice and wildtype controls were subjected to transient brain ischemia by 30 min filamentous middle cerebral artery occlusion (MCAo)/reperfusion.

**Results:**

Acute lesion sizes as assessed by MR-imaging on day 3 did not differ between genotypes. Unexpectedly, however, partial loss of VE-cadherin resulted in long-term stroke protection measured histologically on day 28. Equally surprisingly, VEC^+/-^ mice displayed no differences in post-stroke angiogenesis compared to littermate controls, but showed increased absolute regional cerebral blood flow in ischemic striatum at four weeks. The early induction of VE-cadherin mRNA transcription after stroke was reduced in VEC^+/-^ mice. By contrast, N-cadherin and β-catenin mRNA expression showed a delayed, but sustained, upregulation up to 28 days after MCAo, which was increased in VEC^+/-^ mice. Furthermore, partial loss of VE-cadherin resulted in a pattern of elevated ischemia-triggered mRNA transcription of pericyte-related molecules α-smooth muscle actin (α-SMA), aminopeptidase N (CD13), and platelet-derived growth factor receptor β (PDGFR-β).

**Conclusions:**

Partial loss of VE-cadherin results in long term stroke protection. On the cellular and molecular level, this effect appears to be mediated by improved endothelial/pericyte interactions and the resultant increase in cerebral blood flow. Our study reinforces accumulating evidence that long-term stroke outcome depends critically on vascular mechanisms.

## Background

The concept of the neurovascular unit is proving to be very useful in efforts to elucidate the pathobiology of stroke [1]. Brain ischemia is a vascular disorder affecting neuronal function. Indeed, the biological effects of ischemia are not confined to neurons, but impact an ensemble of different cell types in the brain, which, together, form the neurovascular unit. The bimodal response of the neurovascular unit to stroke is characterized by blood-brain-barrier disruption followed later by vascular remodeling and, hence, the switch from brain injury to repair and recovery.

Endothelia form a critical component of the neurovascular unit. Junctional proteins mediating homotypic interendothelial cell-cell contacts or heterotypic interactions with perivascular cells shape endothelial cell behavior [2]. VE-cadherin is the chief constituent of adherens junctions. Expression of VE-cadherin is strictly confined to endothelia [3]. By contrast, N-cadherin, the second major endothelial cadherin, is also expressed by other cells including neurons [4], astrocytes [5], and pericytes [6].

So far, surprisingly little experimentation has been done on the role of junctional proteins in cerebrovascular disease. Importantly, the primary focus of this research has been on acute blood-brain barrier protection and maintenance of vascular integrity (e.g. [7–9]). However, a number of studies conducted both in human stroke patients and in experimental rodents have demonstrated that acute lesion size is a poor predictor of overall stroke outcome [10]. It should also be noted that endothelial junctional proteins represent interesting candidates for further research because they are potentially druggable (e.g. [11, 12]).

In the current study, we investigated the effects of partial loss of VE-cadherin in VEC^+/-^ mice on both acute and chronic stroke outcome. Acute stroke outcome did not differ between VEC^+/-^ mice and controls. However, chronic lesion sizes were significantly reduced in VEC^+/-^ animals. This was associated with an increase in the overall pattern of mRNA transcription of other junctional proteins and pericyte-related molecules in VEC^+/-^ mice. Taken together, our results suggest that junctional proteins may be harnessed to promote blood flow and recovery after stroke.

## Methods

### Animals and drug treatment

Young adult (12-16 weeks old) male VEC^+/-^ mice and littermate controls were provided by IFOM, Milan, Italy. The generation of these mice has been described previously [13]. The VEC^+/-^ mice were maintained on a mixed genetic background of 129SVJ and CD1 strains [14]. Measurements of body weight before the MCAo procedure were as follows [mean ± S.E.M., range]: VEC^+/+^, 40.8 ± 0.6 g, 33.2 - 48.8 g; VEC^+/-^, 40.3 ± 0.4 g, 35.6 - 45.7 g. Animals were kept under specific pathogen free (SPF) conditions and regularly screened for infections according to FELASA protocols [15]. Mice were maintained on a 12 h light/dark cycle and given ad libitum access to food and water. All animals were acclimated for at least 1 week before surgery. All efforts were made to minimize the number and suffering of animals used. All experimental procedures were approved by the respective official committees (Charité-Universitätsmedizin Berlin and governmental regulatory authority of the federal state) and carried out in strict accordance with the Animal Welfare Act, the European Communities Council Directive of November 24, 1986 (86/609/EEC) and the ARRIVE (Animals in Research: Reporting In Vivo Experiments) guidelines [16].

### Model of cerebral ischemia

Mice were subjected to 30 min left filamentous middle cerebral artery occlusion/ reperfusion as described in detail previously [17–19].

### Magnetic resonance imaging

T2-weighted images at 7 T (Pharmascan; Bruker Biospin, Ettlingen, Germany) were obtained using a fat suppressed two-dimensional turbo spin-echo sequence (repetition time: 5109 msec; echo time: 65.2 msec). A 2 × 2 cm field of view, a 128 × 128 matrix, an in-plane resolution of 156 μm, and a slice thickness of 0.4 mm with no interslice distance were realized [18]. Data acquisition and image processing were carried out with the Bruker software Paravision 4.0. Calculation of lesion volume was carried out with Analyze 10.0 (AnalyzeDirect, Inc.; Lenexa USA). The hyperintense ischemic areas in T2-weighted images were assigned with a region of interest tool. This enables a threshold-based segmentation by connecting all pixels within a specified threshold range about the selected seed pixel and results in a 3D object map of the whole stroke region. The total volume of the whole object map was automatically calculated.

### Immunohistochemistry and immunofluorescence

All procedures have been described in detail previously [20]. Briefly, after an overdose of anesthetics, animals were transcardially perfused with physiological saline followed by 4 % PFA in 0.1 M phosphate buffer, pH 7.4. Brains were dissected from the skulls and postfixed overnight. Before sectioning from a dry ice-cooled copper block on a sliding microtome (Leica), brains were transferred to 30 % sucrose in 0.1 M phosphate buffer, pH 7.4. Brains were cut in the coronal plane in 40 μm thick sections. Sections were stained free floating. Immunohistochemistry followed the peroxidase method with a biotinylated secondary antibody (1:500; Jackson ImmunoResearch Laboratories, West Grove, PA), ABC Elite reagent (Vector Laboratories, Burlingame, CA) and diaminobenzidine (DAB; Sigma) as chromogen. For immunofluorescence, FITC- and RhodX-conjugated secondary antibodies (Jackson ImmunoResearch Laboratories, West Grove, PA) were all used at a concentration of 1:250. Primary antibodies were applied in the following concentrations: anti-NeuN (mouse, 1:100; Chemicon, Temecula, CA), anti-desmin (mouse, 1:100, #6322, Abcam), anti-Glucose Transporter-1 (anti-Glut-1; rabbit, 1:100, #400060, Calbiochem).

### Confocal microscopy

Confocal laser scanning microscopy was performed using a spectral confocal microscope (LSM 700, Zeiss). Appropriate gain and black level settings were determined on control slices stained with secondary antibodies alone.

### Density of perfused vessels

Measurements followed a previously published protocol [21]. Briefly, endovascular dye Evans blue (Sigma-Aldrich; 2 % in saline) was administered intravenously and allowed to circulate for 5 min. Animals were decapitated and brains cut into 10 μm coronal cryostat sections and digitized with a cooled CCD-camera (CoolSNAP EZ, Photometrics, Tucson, AZ) which was attached to a fluorescence microscope. Images of whole brain sections at microscopic resolution were obtained by joining together single camera images using tiled-field mapping software (MCID Elite, InterFocus, Mering, Germany) [21, 22].

### Measurement of cerebral blood flow

Regional absolute cerebral blood flow (CBF) was quantified using the ^14^C-iodoantipyrine technique as described in detail previously [23].

### Dissection of brain tissue

For the molecular analyses, a 4 mm coronal section was dissected from the left (i.e. ischemic) hemisphere (approximately from +5.8 mm to +1.8 mm from interaural line) and stored at -80 °C until further use.

### mRNA isolation and polymerase chain reactions

Tissue was homogenized and total RNA extracted using TRIZOL Reagent (Invitrogen, Karlsruhe). For PCR amplification, we used gene-specific primers (Table 1) and Light Cycler FastStart DNA Master SYBR Green I (Roche Diagnostics, Mannheim). PCR conditions were as follows: preincubation 95 ° C, 10 min; 95 ° C, 15 s, primer specific annealing temperature, 10 s, 72 ° C, 15 s (45 cycles). Crossing points of amplified products were determined using the Second Derivative Maximum Method (Light Cycler Version 3.5, Roche). Quantification of mRNA expression was relative to tripeptidyl peptidase (Tpp) 2 [24]. Specificity of PCR products was checked using melting curve analysis and electrophoresis in a 1.5 % agarose gel.

**Table 1:** Primer sequences used in quantitative real-time PCR

Gene	Sense	Antisense
Tpp2	CTTCTATCCAAAGGCTCTCAAGG	CTCTCCAGGTCTCACCATCATG
VE-cadherin	GCAATGGCAGGCCCTAACTTTC	CAGCAAACTCTCCTTGGAGCAC
N-cadherin	CAGCGCAGTCTTACCGAAGGATG	GCTTCTCACAGCATACACCGTGC
occludin	GAAAGTCCACCTCCTTACAGACC	CATAGCCTCTGTCCCAAGCAAG
claudin-5	CTTCCTGGACCACAACATCGTG	CACCGTCGGATCATAGAACTCG
α-catenin	GAGCCAGTTTCTCAAGGAGGAG	CTGCCATGTCAGCCAGAATCAG
β-catenin	GATGTTGACACCTCCCAAGTCC	CTCATCTAGCGTCTCAGGGAAC
α-SMA	CAGACATCAGGGAGTAATGGTTG	GCTCGTTATAGAAAGAGTGGTGC
CD13	GAAGGCCATGTTCAACATCACAC	GATTCCAATCTGGACACCATTGG
PDGFR-β	CTTTGTGCCAGATCCCACCATG	CTGGAGGCTGTAGACGTAGTAAG

### Statistical analysis

Experiments were carried out in a blinded fashion. Data are presented as means ± S.E.M. Unless otherwise indicated, groups were compared by analysis of variance (ANOVA) with level of significance set at 0.05 and two-tailed *p* values.

## Results

### Expression of adhesion molecules after brain ischemia

**Fig. 1:**
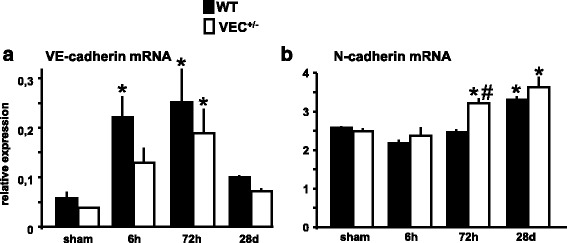
Expression of adhesion molecules after 30 min MCAo/reperfusion. Experiments investigating mRNA expression (also Figs. 2 and 3 a, b, c) were performed in the same animals. Briefly, mRNA expression of VE-cadherin (**a**) and N-cadherin (**b**) was measured in ischemic brain tissue of wildtype and VE-cadherin heterozygous mice after 30 min MCAo/reperfusion or sham operation. 2 VEC^+/+^ mice and 2 VEC^+/-^ animals assigned to the 72 h group died. Furthermore, 3 VEC^+/+^ and 5 VEC^+/-^ mice assigned to the 28 d group died. The resultant group sizes were as follows: 3 VEC^+/+^ sham, 3 VEC^+/-^ sham, 4 VEC^+/+^ 6 h, 4 VEC^+/-^ 6 h, 4 VEC^+/+^ 72 h, 4 VEC^+/-^ 72 h, 6 VEC^+/+^ 28 d, 4 VEC^+/-^ 28 d. Relative mRNA expression is reported as the value normalized to tripeptidyl peptidase 2 (Tpp2) for each sample. One-way ANOVA, *p < 0.05 versus sham, #*p* < 0.05 between genotypes within time point

First, we measured mRNA expression of VE- and N-cadherin in ischemic brain tissue of wildtype and VE-cadherin heterozygous mice in the sham condition as well as at different time points after 30 min MCAo/reperfusion. Note that VE-cadherin mRNA levels are markedly reduced, but by no means completely depleted, in VEC^+/-^ mice (Fig. 1a). In both wildtype and VEC^+/-^ mice, VE-cadherin mRNA levels rose transiently shortly after brain ischemia (Fig. 1a). Interestingly, the mRNA expression of N-cadherin, showed a delayed upregulation after MCAo. This effect was significantly increased in VEC^+/-^ mice compared to the wildtype mice at 72 h (Fig. 1b).

### Expression of junctional molecules after brain ischemia is influenced by VE-cadherin

**Fig. 2:**
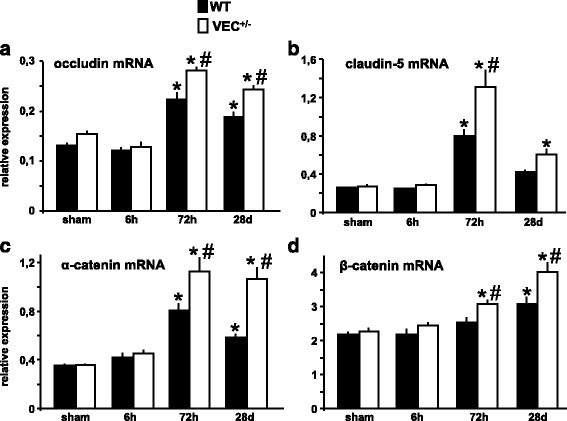
Expression of junctional molecules after brain ischemia is influenced by VE-cadherin (**a-d**) mRNA expression of key junctional molecules occludin, claudin-5, α-catenin and β-catenin was assessed in ischemic brain tissue of wildtype and VE-cadherin heterozygous mice after 30 min MCAo/reperfusion or sham operation. Relative mRNA expression is described as the value normalized to tripeptidyl peptidase 2 (Tpp2) for each sample. *N* = 3-6 animals per group. One-way ANOVA, *p < 0.05 versus sham, #*p* < 0.05 between genotypes within time point

Furthermore, the mRNA expression of key junctional molecules occludin, claudin-5, α-catenin and β-catenin was assessed. Again, the mRNA expression of these junctional molecules showed a delayed, yet sustained upregulation after MCAo. This effect was generally more pronounced in VEC^+/-^ mice (Fig. 2a-d).

### Partial loss of VE-cadherin alters the response of pericyte-associated molecular signatures to brain ischemia

**Fig. 3:**
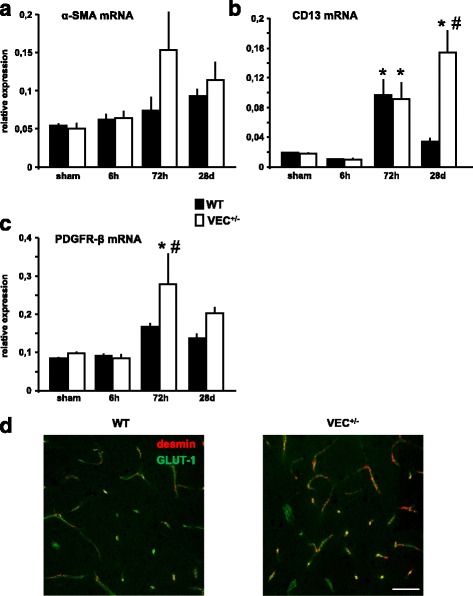
Expression of pericyte markers after 30 min MCAo/reperfusion. **a-c** mRNA transcription of pericyte-related molecules was measured in ischemic brain tissue of wildtype and VE-cadherin heterozygous mice after 30 min MCAo/reperfusion. Relative mRNA expression is reported as the value normalized to tripeptidyl peptidase 2 (Tpp2) for each sample. *N* = 3-6 animals per group. One-way ANOVA, *p < 0.05 *versus* sham, #*p* < 0.05 between genotypes within time point. α-SMA (alpha-smooth muscle actin), CD13 (aminopeptidase N), PDGFR-β (platelet derived growth factor receptor-β). **d** Increased pericyte coverage of vasculature in the ischemic brain of VEC^+/-^ mice at 28 days after MCAo. Green: GLUT-1. Red: desmin. Scale bar 50 μm

Pericytes are commonly identified by a combination of molecular markers such as α-smooth muscle actin (α-SMA), aminopeptidase N (CD13), and platelet-derived growth factor receptor β (PDGFR-β). Overall, we found a pattern characterized by increased ischemia-induced mRNA transcription of pericyte-related molecules in VEC^+/-^ mice (Fig. 3a-c). Remarkably, CD13 mRNA levels in VEC^+/-^ mice remained strongly elevated even four weeks after MCAo, whereas in wildtype mice, transcription of CD13 reverted back to baseline concentrations (Fig. 3b). Based on these findings, desmin immunoreactivity was used to visualize pericyte coverage of Glut-1 expressing endothelial cells (Fig. 3d) in the ischemic MCA territory [25]. In line with the mRNA data, pericyte coverage in VEC^+/-^ mice was noticeably increased.

### Absolute cerebral blood flow in ischemic striatum is elevated in VEC^+/-^ mice at four weeks after brain ischemia

**Fig. 4:**
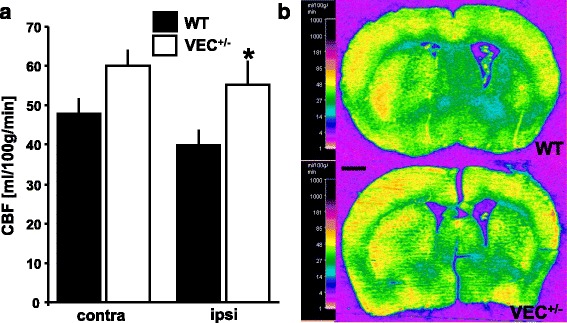
Measurement of absolute cerebral blood flow (CBF) in ischemic striatum. **a** CBF was measured in ischemic striatum (ipsi) and the corresponding area of the contralateral hemisphere (contra) at four weeks after 30 min MCAo using the 14C-iodoantipyrine tissue equilibration technique. The experiments depicted here as well as in Figs. 5 and 6a were conducted in the same animals. Briefly, of 10 VEC^+/+^ and 16 VEC^+/-^ mice entered into the experiment, 2 VEC^+/+^ and 5 VEC^+/-^ mice died after MCAo. One further VEC^+/+^ mouse died during preparation for blood flow experiments. Moreover, due to technical issues with the femoral artery catheter, one VEC^+/+^ and one VEC^+/-^ animal each could not be used for measurement of CBF. The analysis presented here is therefore based on 6 VEC^+/+^ and 10 VEC^+/-^ mice. Finally, the ipsilateral side of the brain of one further VEC^+/-^ mouse sustained substantial damage during cryostat sectioning, so the ipsilateral hemisphere of this animal is also not included here. Two-way ANOVA revealed an overall effect of factor ‘genotype’ on CBF. Newman-Keul’s post hoc test, *p < 0.05 between genotypes within the ischemic striatum. Representative pseudocolored autoradiographic images of coronal brain sections are shown in (**b**). Scale bar in (**b**) 1 mm

Absolute cerebral blood flow (CBF) was measured at four weeks after 30 min MCAo using the ^14^C-iodoantipyrine tissue equilibration technique. Analysis of blood flow was performed in the ipsilateral (i.e., ischemic) and contralateral striatum. Surprisingly, absolute CBF in the ischemic striatum of VEC^+/-^ mice was significantly higher than CBF in ischemic striatum of wildtype controls (Fig. 4a, b). Physiological parameters including body weight, mean arterial blood pressure, heart rate and arterial blood gases did not differ between genotypes (Table 2).

**Table 2:** Physiological data

	Weight (g) before MCAo	Weigth (g) before CBF	MABP (mmHg)	Heart rate (n/min)	pH	pCO2 (mmHg)	pO2 (mmHg)
WT	43.2 ± 1.7	41.9 ± 1.6	94 ± 1.7	366 ± 10	7.28 ± 0.02	42.7 ± 0.7	94.8 ± 2.0
VEC^+/-^	41.6 ± 0.9	38.8 ± 0.6	100.3 ± 2.3	391 ± 7.8	7.29 ± 0.01	44.1 ± 0.7	95.6 ± 1.3

Summary of physiological data obtained during CBF measurements. Weight (g) before MCAo: Body weights of all mice entered into the CBF experiments (10 VEC^+/+^ and 16 VEC^+/-^). Weight (g) before CBF: Body weights of all mice which had survived up to this point (*N* = 8 VEC^+/+^ and 11 VEC^+/-^ mice). MABP (mmHg), heart rate (n/min), pH, pCO2 (mmHg), and pO2 (mmHg) were also assessed in 8 VEC^+/+^ mice and 11 VEC^+/-^ mice. Please note that, following physiological measurements, one VEC^+/+^ mouse died during preparation for blood flow experiments. Moreover, due to technical issues with the femoral artery catheter, one VEC^+/+^ and one VEC^+/-^ animal each could not be used for measurement of CBF (see legend to Fig. 4)

### Partial loss of VE-cadherin does not affect neovascularization at four weeks after MCAo

**Fig. 5:**
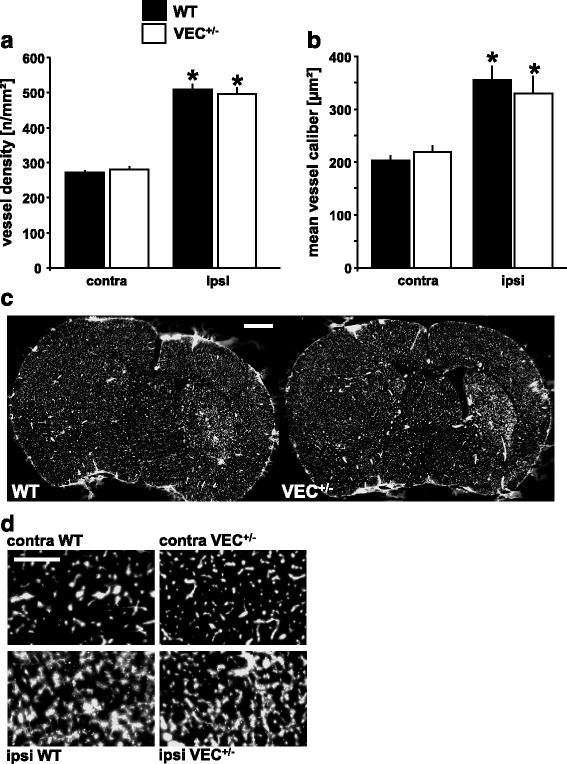
Neovascularization in ischemic striatum. Density (**a**) and mean caliber (**b**) of Evans blue-filled vessels were determined in ischemic striatum (ipsi) and corresponding area of contralateral hemisphere (contra) using tiled-field mapping and computer-assisted image analysis. Representative examples of Evans blue tiled-field images at low- (**c**) and high-power magnification (**d**). Note significant inducing effect of ischemia, but not of genotype, on vessel density and mean vessel caliber in wildtype mice and in VEC^+/-^ mice at four weeks after 30 min MCAo/reperfusion. The experiments depicted here as well as in Figs. 4 and 6a were conducted in the same animals. Briefly, of 10 VEC^+/+^ and 16 VEC^+/-^ mice entered into the experiment, 2 VEC^+/+^ and 5 VEC^+/-^ mice died after MCAo. One further VEC^+/+^ mouse died during preparation for blood flow experiments. Finally, the ipsilateral side of the brain of one further VEC^+/-^ mouse sustained substantial damage during cryostat sectioning, so this animal could also not be included in this analysis. *N* = 7 VEC^+/+^ and *n* = 10 VEC^+/-^ animals per group. Two-way ANOVA followed by Newman-Keul’s post hoc test. *p < 0.05 relative to contralateral (i.e. non-ischemic) striatum within each genotype. Scale bar in (**c**) 1 mm (both images), scale bar in (**d**) 200 μm (all four images)

The density of perfused microvessels was quantified at 28 days after MCAo/reperfusion using endovascular Evans blue staining (Fig. 5a-d). The density of perfused microvessels was significantly increased in ischemic as compared to contralateral striatum. Furthermore, brain ischemia led to alterations of microvascular structure in ischemic brain tissue with significantly enlarged vessels. However, in seeming contradiction to the results of absolute CBF measurements described above, neither vessel density nor mean vessel caliber differed significantly between VEC^+/-^ mice and wildtype controls.

### Partial loss of VE-cadherin confers reduced lesion sizes at four weeks after MCAo

**Fig. 6:**
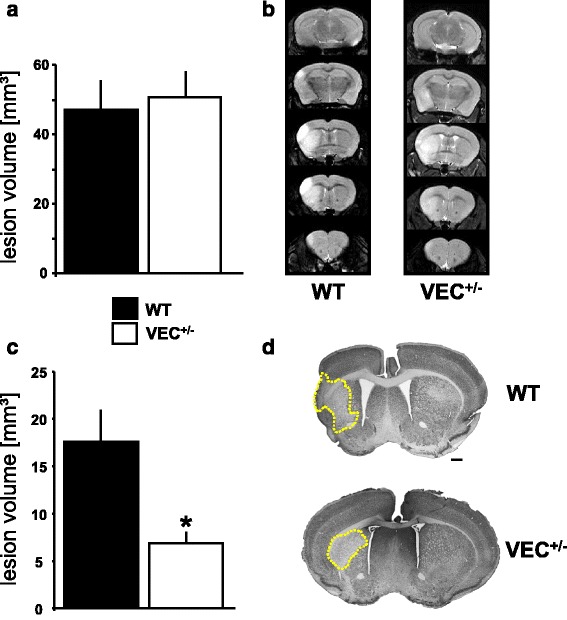
Acute and chronic lesion sizes. **a**, **b** The experiments depicted in Figs. 4 and 5 as well as here in Fig. 6a were conducted in the same animals. Of 10 VEC^+/+^ and 16 VEC^+/-^ mice entered into the experiment, 2 VEC^+/+^ and 4 VEC^+/-^ mice died shortly after MCAo. **a** Acute neuronal damage was assessed at 72 h after 30 min MCAo/reperfusion using 7 T MRI. One further VEC^+/+^ mouse had to be excluded because, due to technical problems, MRI images in this animal were not of sufficient quality to allow a quantitative analysis. Therefore, the statistical evaluation is based on 7 VEC^+/+^ and 12 VEC^+/-^ mice. T-test, *p < 0.05 versus wildtype. Representative MR-images (T2) are given in (**b**). **c**, **d** Chronic stroke outcome was assessed using NeuN immunohistochemistry. Direct cerebral lesion volumes (**c**) were determined on five coronal brain sections (approximately interaural 6.6 mm, 5.3 mm, 3.9 mm, 1.9 mm and -0.1 mm) by computer-assisted volumetry. Here, of 10 VEC^+/+^ and 10 VEC^+/-^ mice entered into the experiment, 4 VEC+/+ and 3 VEC+/- survived to the time of histological analysis at four weeks after MCAo/reperfusion. T-test, *p < 0.05 versus wildtype. Representative images of NeuN immunohistochemistry are presented in (**d**). Scale bar in (**d**) 500 μm

Acute structural damage after brain ischemia was assessed at 72 h using MR imaging (Fig. 6a, b). At this early time point, lesion volumes were similar in VEC^+/-^ mice and wildtype controls. However, when chronic stroke outcome was evaluated histologically on day 28, a significant difference between genotypes with reduced stroke volumes in VEC^+/-^ mice had emerged (Fig. 6c, d).

## Discussion

Stroke is a vascular disorder affecting neuronal function. Notwithstanding, the bulk of experimental stroke research has so far focussed rather one-sidedly on mechanisms to salvage neuronal cells and may thus have contributed to the apparent failure of bench-to-bedside translation plaguing the field in recent years (e.g. [26]). However, stroke affects different cell types in the brain, which conjointly form the so-called ‘neurovascular unit’. For instance, in the gray matter of the human cerebral cortex, non-neuronal cells outnumber neurons by a factor of approximately 1 to 2 [27]. In consequence, brain ischemia impacts not only neurons but also astrocytes and other glial cells that support the neurons, the axons of neurons that relay their signals to other cells, and finally, and very importantly, the microvessels that supply oxygen and nutrients to them. These considerations provide a firm basis for shifting the previous focus from neurons alone to the complex of neurons, microvessels and supportive cells (astrocytes, pericytes and resident inflammatory cells) reacting to brain ischemia. Conceptually, the response of the neurovascular unit to brain ischemia may be divided into two distinct phases: (i) damage to the neurovascular unit during the acute phase of stroke is followed by (ii) a phase of regeneration and partial restoration of function. The detailed mechanisms that govern this switch from early injury to delayed recovery are still poorly understood.

Endothelial cells are a crucial component of the neurovascular unit. Early on, endothelial cell injury results in blood-brain barrier dysfunction, which favors tissue swelling and may lead to intracerebral hemorrhage after stroke (e.g. [28]). At later stages, the formation of new vessels with altered morphology and microvascular structure has been observed in ischemic brain tissue [29].

Importantly, a number of studies, both by us and others, have established the notion that vascular remodeling after stroke is a critical determinant of chronic stroke outcome (e.g. [19, 21, 30, 31]).

This study yields a number of new and unexpected insights into the vascular biology of ischemic stroke. In a nutshell, we show that the N-cadherin/VE-cadherin balance can be manipulated such that overall stroke outcome is improved. Contrary to our initial assumption, partial loss of VE-cadherin did not confer an increase in acute lesion size at 72 h. However, none of the junctional proteins investigated including VE-cadherin itself showed a significant difference in mRNA expression between genotypes at baseline. Furthermore, brains from heterozygous mice appeared phenotypically normal, so the genotypic differences were likely not large enough to translate into an early phenotypic difference after MCAo. Furthermore, we also did not detect significant differences in microvessel densities or absolute regional cerebral blood flow in the non-ischemic contralateral hemispheres between VEC^+/-^ mice and wildtype controls.

More interestingly still, our results demonstrate that reduced VE-cadherin expression leads to improved vascular remodeling and a reduction in chronic lesion sizes after stroke. In how far these beneficial effects of reduced VE-cadherin expression also promote functional rehabilitation after stroke (i.e. neuro-behavioral assessment) lies beyond the scope of the present work. The following points in particular, for our purposes, merit further discussion here. Our finding of increased N-cadherin and β-catenin mRNA expression in VEC^+/-^ mice after stroke recapitulates earlier findings on endothelial cells in development, where N-cadherin mRNA and protein expression was reduced by VE-cadherin via inhibition of β-catenin signaling [14]. N-cadherin, in turn, recruits pericytes, which also express N-cadherin, to endothelial cells [6, 14, 32]. Accordingly, we investigated mRNA transcription of a panel of pericyte markers. Although not corroborated at the protein level, we detected a compelling pattern of elevated mRNA levels of pericyte-related molecules after MCAo. On a related note, it is also worth mentioning that the density of perfused microvessels after stroke did not differ between VEC^+/-^ mice and wildtype controls, but absolute cerebral blood flow did. In line with these seemingly contradictory observations, an in vivo model of brain angiogenesis in the absence of pericytes also yielded normal microvessel density, but abnormalities in microvessel architecture [33]. It has to be acknowledged that, for this investigation, we did not assess CBF in separate sham controls of either genotype but limited ourselves to the analysis of blood flow in the contralateral striatum, which served as an internal control. Brain ischemia is a strong angiogenic stimulus [19, 21]. Our study of post-stroke vascular remodeling thus reinforces recent findings indicating that endothelial/pericyte interactions are central processes in the regulation of vessel formation, stabilization, remodeling, and function [34]. Importantly, pericytes are also beginning to emerge as major regulators of cerebral blood flow [35, 36]. Targeting endothelial/pericyte interactions through junctional proteins may therefore provide a new avenue for stroke treatment especially during the subacute phase of recovery.

## Conclusions

To summarize, our study reinforces accumulating evidence that long-term stroke outcome depends critically on vascular mechanisms and, in particular, cerebral blood flow. Endothelial junctional proteins may be a potent target for stroke treatment fostering regeneration during the subacute phase of recovery.

## Abbreviations

CBF: Cerebral blood flow
CD13: Aminopeptidase N
MCAo: Middle cerebral artery occlusion
PDGFR-β: Platelet-derived growth factor receptor β
VEC^+/-^: VE-cadherin heterozygous
α-SMA: α-smooth muscle actin.
